# Unexpected varus deformity and concomitant metal ion release and MRI findings of modular-neck hip stems: descriptive RSA study in 75 hips with 8 years’ follow-up

**DOI:** 10.1080/17453674.2020.1853387

**Published:** 2020-12-10

**Authors:** Sverrir Kiernan, Bart Kaptein, Carl Flivik, Martin Sundberg, Gunnar Flivik

**Affiliations:** a Department of Orthopedics, Skåne University Hospital, Clinical Sciences, Lund University , Lund , Sweden ;; b Department of Orthopedics, Leiden University Medical Centre , Leiden , The Netherlands

## Abstract

Background and purpose — Modular-neck hip stems have been identified with corrosion-related problems from the neck–stem junction. We report an ongoing varus deformity of modular-neck hip stems with simultaneous metal ion release observed during a study comparing the migration of modular vs. standard hip stems.

Patients and methods — We followed 50 patients with modular and 25 with standard neck stems using radiostereometry (RSA). At 5-year follow-up, we noted a compromised integrity of the modular stem with varus deformity in the neck–stem interface. Changes in head–tip distance as well as whole-blood ion concentration and MRI findings were analyzed. The modular stems were followed further up to 8 years.

Results — The head–tip distance decreased continuously by 0.15 mm per year resulting in 1.2 (95% CI 1.0–1.4) mm at 8 years for modular stems, while for the standard stems at 5 years, the decrease was 0.09 (CI 0.0–0.2) mm or 0.02 mm/year. For the modular stems, the reduction in head–tip distance correlated to the increase in whole-blood cobalt concentration at 8 years but not to the MRI grading of tissue reactions. At 5 years, cobalt levels were 4.9 µg/L for modular stems and at 8 years 4.8 µg/L, whereas for standard stems this was 1.0 µg/L. After 8 years, 9 of 72 stems had been revised for different reasons, but only 1 with obvious adverse local tissue reaction (ALTR).

Interpretation — We present a surprisingly large progressive deformation at the modular neck–stem junction, but so far without a definite clinical problem. Even the femoral head seems to show slight compression onto the taper over time. A high rate of revisions for the modular type of this stem has raised general concerns, and it has been recalled from the market.

Modular hip stems with different versions, angles, and lengths of neck can adapt to different femoral geometries. These modifications are valuable theoretically for improving range of motion and soft tissue balance (Barrack 1994, Jones 2004, Archibeck et al. 2011, Srinivasan et al. 2012).

Mechanically assisted crevice corrosion (MACC) became clinically relevant with the emergence of large metal-on-metal (MoM) implants early in the 21st century (McGrory and McKenny 2016). Recently, corrosion has also been reported for metal-on-polyethylene (MoP) in a variety of stem designs caused by fretting in the head–neck junctions (Gilbert et al. 1993, Morlock 2015, Patel et al. 2016, Morlock et al. 2018). The increased number of interfaces introduced by modular systems has the potential to increase the risk for adverse local tissue reaction (ALTR) caused by the release of metal ions and inflammatory mediators (Molloy et al. 2014, McGrory and McKenney 2016). Taper corrosion at the modular junctions of THA femoral stems are known to cause ALTR (Lindgren et al. 2011, Gill et al. 2012). Revisions as a consequence of ALTR associated with neck–stem taper corrosion have now been reported (Nahhas et al. 2019, Shah et al. 2019) and should be considered as a potential cause for progressive, disabling groin pain (Cooper et al. 2013).

The cause of corrosion in the neck–stem junction has been debated. Some say that the shape of the neck–stem tapers may deviate from ideal dimensions causing relative motions between the neck and stem (Frisch et al. 2018). Others state that corrosion occurs regardless of design and that the primary cause is mixed-metal couples with unequal modulus of elasticity (Young’s modulus), allowing for increased metal transfer and surface damage (galvanic mode of corrosion) (Su et al. 2017).

In 2010, we started a randomized controlled trial (Kiernan et al. 2020) to evaluate the potential superiority of modular stems in restoring hip symmetry and examine postoperative migration rates of ABG II modular vs. standard stems (ABG II system, Stryker, Exeter, UK) with RSA (Figure 1). During the follow-up and data-processing, we achieved some unexpected results regarding the modular design. At the 5-year follow-up, we noted that the complex consisting of the stem–neck–head as a rigid body used for RSA was no longer a fixed segment, and the prosthetic head seemed to have migrated with respect to the body of the stem. To investigate this phenomenon, we decided to measure the movement of the head in relation to the tip point of the stem. To find out if it might be due to corrosion and fretting in the neck–stem junction of the modular stem, we correlated the rate of deformation with whole–blood ion levels of cobalt, chromium, and titanium. We measured ALTR formation based on Metal Artefact Reduction Series MRI (MARS-MRI) and analyzed whether stem size, neck length, head length, caput-collum diaphyseal (CCD) angle, version, total neck length, and body weight influenced this deformation of the stem complex.

**Figure 1.**
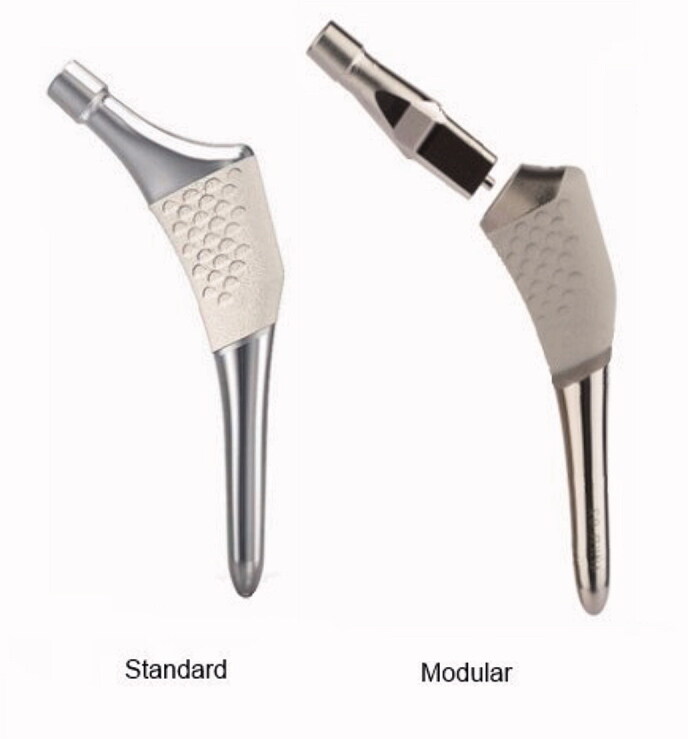
Standard and modular stem designs used in this study.

## Patients and methods

Of the 75 stems that we had in our cohort for the former ABG II study (Kiernan et al. 2020), we were able to include 47 modular stems with sufficient RSA data during the follow-up period and 25 non-modular standard stems (up to 5 years’ follow-up). We lost 1 patient to follow-up due to an early periprosthetic femoral fracture (PPFF); we excluded 2 because of problems in performing the postoperative RSA examination (Table 1).

**Table 1.** Demographic and treatment data of study population

	Modular	Standard
Factor	(n = 47)	(n = 25)
Male/female sex	30/17	16/9
Age (range)	58 (34–80)	60 (46–74)
BMI (SD)	28 (3.8)	28 (4.0)
Weight, kg (SD)	84 (13)	88 (17)
Components used		
Size, mean (range)	5 (1–7)	6 (4–8)
Short/long neck	23/24	
Retroverted/standard/anteverted neck	16/19/12	
CCD angle (125°/130°/135°)	37/6/4	
Head length, (–5/standard/+5)	10/30/7	12/11/2
Total neck length, mm median (range)	58 (47–69)	58 (53–65)

The 47 modular hips were studied in further detail to reveal the influence of stem size, neck length (short/long), neck angle (125°/130°/135°), head length (–5 mm/standard/+ 5mm), neck version (anteverted, standard, retroverted), and patient body weight. We also looked at the total neck length (combined neck and head length) and correlated it to the HTD reduction over time.

The ABG II prosthesis has a titanium alloy (TMZF) stem. The modular neck is a cobalt-chromium (CoCr) alloy. We used a 36 mm CoCr LFIT femoral head for all patients except 2 modular stems that had 32 mm head due to small-sized cups. We used uncemented cups with a highly crosslinked poly­ethylene liner.

### Radiostereometric analysis

We used a uniplanar technique with the patient supine (Valstar et al. 2005) with 2 fixed X-ray sources. We used a type-41 calibration cage (Tilly Medical, Lund, Sweden) and the model-based RSA software (Version 4.0, RSAcore, Leiden, The Netherlands). The reference examination was performed on the 1st postoperative day before mobilization and served as the reference point for all further examinations. Follow-up examinations were carried out at 14 days, 3 months, and then at 1, 2, and 5 years and additionally for the modular stems at 8 years.

**Figure 2.**
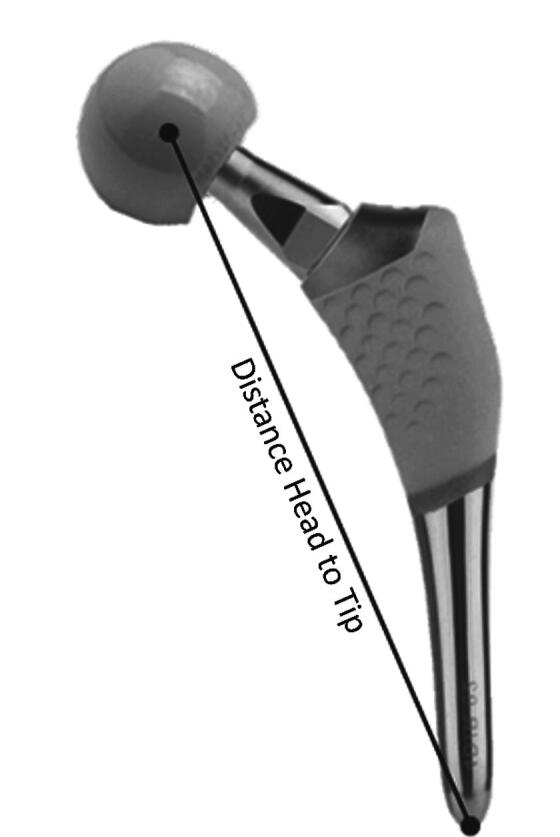
Measurement of the head–tip distance.

We performed model-based RSA analysis using the EGS hip analysis method that includes accurate estimation of the positions of the head and distal tip of the hip stem (Kaptein et al. 2006). Our primary outcome was the change in the distance between the center of rotation of the prosthetic head and the tip of the stem measured by successive RSA using the postoperative examination as reference (Figure 2).

The precision of the RSA measurements for the head–tip distance was 0.15 mm and based on 68 patients’ double examinations. The precision represents the smallest migration value that is considered significant and is based on 2 standard deviations of the error obtained representing the 95% confidence interval.

### Metal ion measurements

We measured the levels of cobalt, chromium, and titanium at 5 years for both stem designs for comparison and additionally at 8-year follow-up for correlation with the rate of stem deformation in patients operated on with the modular stem. Measurements obtained metal ion concentrations in whole blood (SGAB Analytica, Luleå University of Technology, S-971 87 Luleå, Sweden) (Rodushkin et al. 2000).

### ALTR assessment on MARS-MRI

We evaluated all patients who agreed to an MRI at 5 years for the occurrence of ALTR and graded the MARS-MRI findings. 3 patients with modular stems and 1 with standard refused the examination. We used the 1.5 Tesla MRI system (MAGNETOM Avanto, Siemens AG, Healthcare Sector, Erlangen, Germany) using spine matrix and body matrix coils, running a protocol consisting of coronary T1 view angle tilting (VAT) + STIR VAT, sagittal T2 VAT and axial T1 VAT. Intravenous contrast (19 mL Dotarem) was administered, and then an axial T1 VAT together with an axial subtraction image was conducted. This resulted in 6 image sequences. An experienced musculoskeletal radiologist analyzed all MRIs and graded the findings using a modified version of the Hauptfleisch grading system (Hauptfleisch et al. 2012). This system consists of type I: cystic ALTR with a wall thickness < 3 mm; Type II: Cystic ALTR with a wall thickness > 3 mm; Type III: solid ALTR. We added type 0 (no ALTR) and divided type II into subgroups IIa (cystic ALTR without solid parts) and b (with a solid part, but comprising less than 50% of the total ALTR area).

### Measurement of total neck length

The modular design had 3 parts, i.e., a stem, neck, and head. With the standard design, the offset increases with size, while the modular design allows an improved range of motion and soft tissue balance by various choices in neck length, version, and CCD angle.

**Figure 3.**
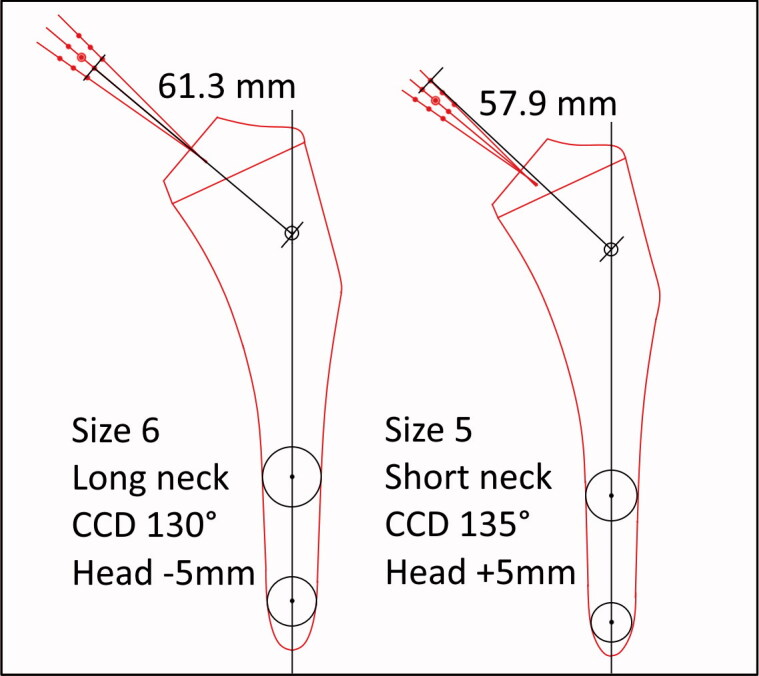
Examples of measurements of total neck length. The figure shows all possible measuring points along the 3 different CCD angles. The point of intersection is defined as the intersection between the CCD 130° line and the longitudinal axis of the stem.

We measured the total neck length of all implanted modular stem combinations for estimation of the effect of the combined lever arm gained by different component choices. We did this by measuring the length of a line from the head center to the point of its intersection with the longitudinal axis of the stem on calibrated templates (Figure 3).

### Clinical evaluation

For clinical evaluation, preoperatively and at 1, 2, and 5-year follow-up, we used the hip-specific Hip Osteoarthritis Outcome Score (HOOS), Swedish version LK 2.0, as well as VAS pain and VAS satisfaction.

### Statistics

We used estimates from a general linear mixed model for the analysis of head–tip distance reduction, where the subject effect was taken into consideration, and estimates from a linear regression model for the analysis of whole-blood ion levels with regard to the rate of stem deformation. All models were specified as the main effect of each predictor and time, and the interaction between them. Both random slopes and intercepts were used and the covariance matrix was specified as an AR-1 correlation. Metal ion levels were modeled with linear regression, where each metal was modeled against movement, respectively. This was done separately for the 5- and 8-year follow-up. All linear mixed models were estimated using the R package nlme (R Foundation for Statistical Computing, Vienna, Austria). The Mann–Whitney U-test was used to compare the distribution for the different grades of ALTR as well as to test for differences between groups for VAS and HOOS. All calculations were conducted in R v.3.5.2 (R Foundation for Statistical Computing, Vienna, Austria).

### Ethics, registration, data sharing, funding, and potential conflicts of interest

The Regional Ethical Review Board at Lund University approved the original study (Dr 2009/6), and it was carried out in compliance with the Helsinki Declaration and registered in ClinicalTrials.gov Identifier: NCT01512550. Data is available on reasonable request.

The Southern Healthcare Region in Sweden (Södra Sjukvårdsregionen) provided a doctoral grant for labor costs. Stryker gave financial support for part of the RSA and MRI examinations but did not influence how we conducted or interpreted the study. The authors declare no conflict of interest related to this study.

## Results

### Revisions

**Figure 4.**
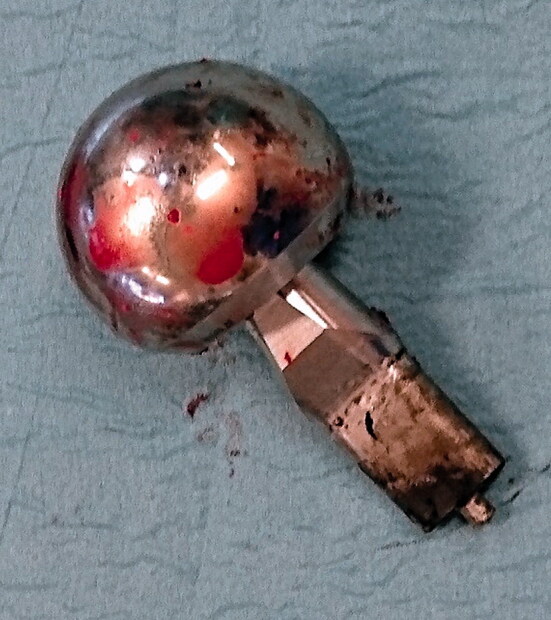
ABG II modular head (LFit) and neck after revision with corrosion on the neck part engaged in the stem–neck junction.

8 modular stems had been revised at the 8-year follow-up, 1 due to hip pain in combination with raised metal ion levels and MRI signs of ALTR. 1 revision was due to loosening of the stem, and 2 revisions due to loosening of the cup where we also decided to revise the well-fixed stems. 3 stems were revised because of periprosthetic femoral fractures (PPFF) with adequate trauma and 1 because of late periprosthetic infection. None of the modular necks showed perioperative signs of loosening from the stem, and they had to be dismounted with force. The metal on both stem and neck junctions showed signs of corrosion with black discoloration (Figure 4). 1 of the hips in the standard group was revised before the 5-year follow-up because of periprosthetic infection (Table 2).

**Table 2.** Time to and cause for revisions

Factor	Years to revision	Cobalt (µg/L)	Chromium (µg/L)	Titanium (µg/L)	ALTR grade ^a^
Modular:					
Infection	1.9	n/a	n/a	n/a	n/a
Loose cup	3.6	3.3	1.0	1.7	n/a
Loose cup	4.3	3.8	1.7	0.5	n/a
PPFF ^b^	5.8	3.0	0.9	2.8	4
PPFF	6.4	5.4	1.9	0.5	0
ALTR ^c^	6.4	8.2	2.4	1.3	3
PPFF	7.1	9.3	1.8	0.5	1
Loose stem	7.8	4.8	1.1	0.5	1
Standard:					
Infection	1.4	n/a	n/a	n/a	n/a

ALTR: Adverse local tissue reaction .

PPFF: Periprosthetic femoral fracture.

**^a^** At 5 years’ follow-up.

**^b^** Accompanying groin pain prior to PPFF.

**^c^** ALTR type 3. Skin reaction with proved hypersensitivity to cobalt. Accompanying groin pain.

### Radiostereometric analysis

**Figure 5.**
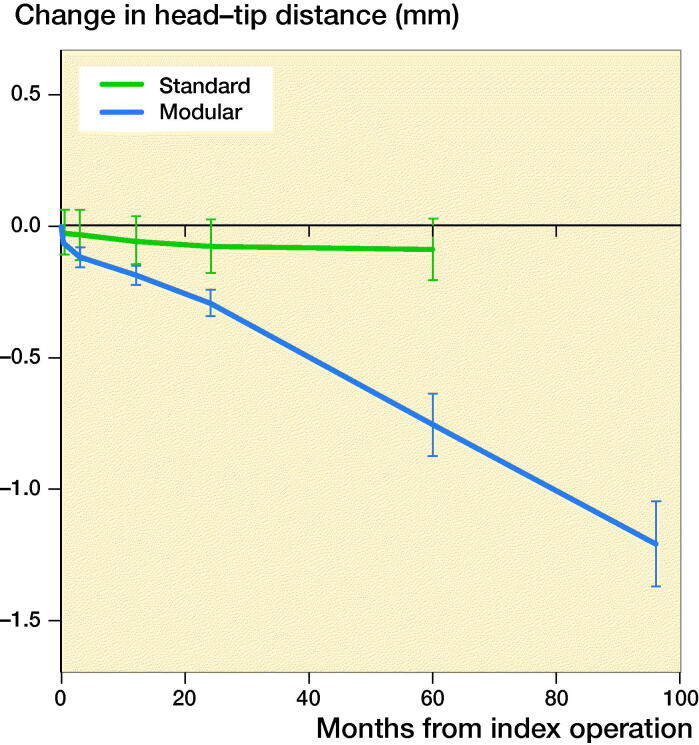
Mean values with 95% CI of the reduction in head–tip distance in mm for different follow-up moments in months up to 5 years for the standard design and up to 8 years for the modular version.

For the modular group, at 5 years, the mean change in HTD was –0.75 mm (range –1.64 to 0.14), equivalent to –0.15 mm/year. For the standard group, the change was –0.09 mm (–1.07 to 0.33) or –0.02 mm/year. We then continued to follow the modular group, and at 8 years the change in HTD was –1.21 mm (–1.94 to –0.10) or still at the same pace of –0.15 mm/year. This HTD reduction was statistically significant over time for the modular group (p < 0.001) but not for the standard group (p = 0.3). There was a statistically significant difference in HTD between the modular and standard groups from the 2nd year follow-up onwards, and by the 5-year follow-up the difference was 0.66 mm (Figure 5). No comparison could be made at 8 years as we followed the standard group for only 5 years.

**Figure 6.**
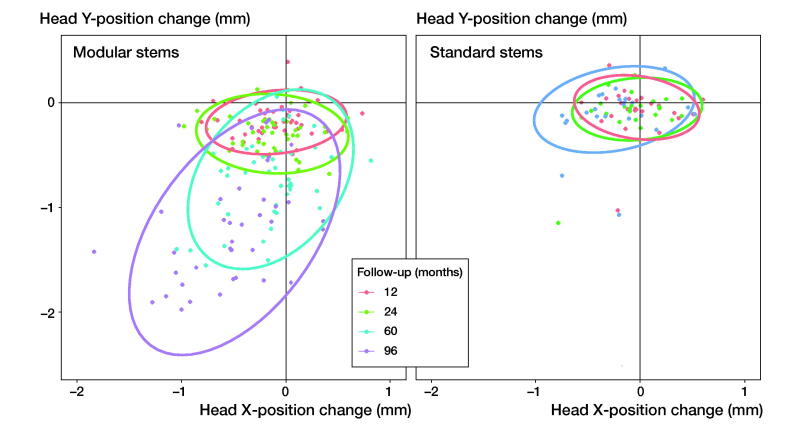
Change in position of the hip head with respect to the postoperative situation in X-direction (perpendicular to the hip–stem axis) and Y-direction (along the hip–stem axis), for 1, 2, 5, and 8 years’ postoperative follow-ups. The ellipse presents the 95% prediction interval of the head position change for each follow-up moment.

The reduction in the HTD was evident in modular stems but not in standard stems (Figure 6).

### Metal ion measurements

We found a difference between standard and modular designs for all metal ion results at 5 years, with higher levels for the modular group (Table 3).

**Table 3.** Metal ion levels at 5- and 8-year follow-up for stem designs. Values are mean µg/L (95% CI)

Factor		5 years	8 years
Modular	Cobalt	4.9 (4.1 − 5.7)	4.8 (4.3 − 5.3)
	Chromium	1.8 (1.5 − 2.0)	1.3 (1.0 − 1.6)
	Titanium	1.3 (1.1 − 1.5)	1.2 (1.0 − 1.5)
Standard	Cobalt	1.0 (0.7 − 1.4)	
	Chromium	0.9 (0.4 − 1.4)	
	Titanium	0.8 (0.6 − 1.0)	

**Figure 7.**
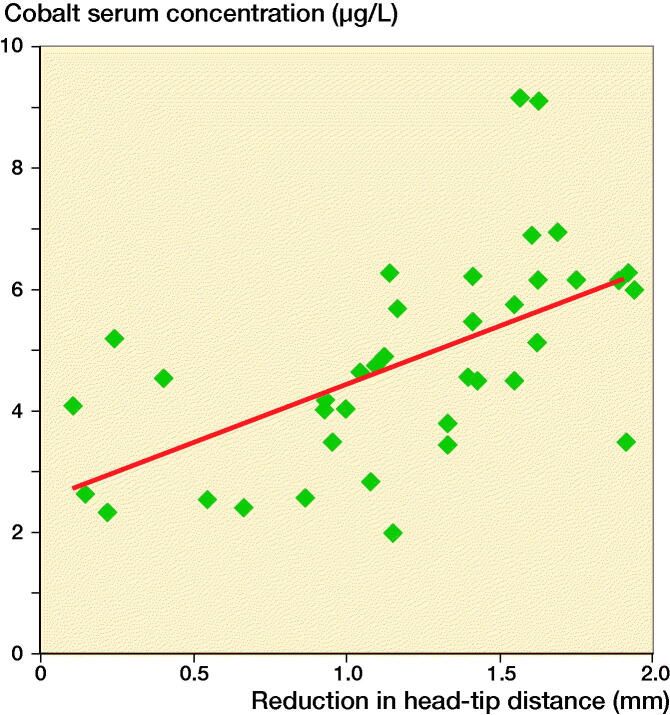
Estimates from our linear regression model showing cobalt whole-blood concentration vs. reduction in head–tip distance at the 8-year follow-up.

According to estimates from our linear regression model for the modular stem, a 1mm reduction in HTD corresponds to a 1.9 µg/L (CI 1.03–2.8) increase in whole-blood cobalt concentration at 8 years (Figure 7). We found no statistically significant reduction in HTD and whole-blood concentration of chromium or titanium (p = 0.4 and 0.6). The metal ion concentrations (Co, Cr, and Ti) leveled out after the 5-year follow-up.

### ALTR assessment on MARS-MRI

There were no differences in ALTR grade between stem designs (Table 4), nor was there any correlation between the level of any of the metal ions and grade of ALTR.

**Table 4.** ALTR grades assessed on MARS-MRI

ALTR grades	Modular (n = 45)	Standard (n = 22)
0	19	13
1	10	2
2	2	0
3	6	1
4	3	5
Missing	5	1

0 = no ALTR; 1 = cystic ALTR with a wall thickness of < 3 mm;

2 = cystic ALTR with a wall thickness of > 3 mm and without any solid parts;

3 = cystic ALTR with a wall thickness of > 3 mm with a solid part, but comprising less than 50% of the total ALTR area;

4 = solid ALTR.

### Modular components used

Stem size, neck length, neck angle, head length, neck version (anteverted, standard, retroverted), sex, and patient body weight did not influence HTD reduction.

We used the median neck length to divide all modular stems into 2 equal groups (long vs. short) (Table 1) and found a mean of 0.3 (CI –0.0 to 0.6) mm greater reduction in HTD in longer total neck lengths.

### Clinical evaluation

Pain and satisfaction were similar between modular and standard stems preoperatively and throughout the 5-year follow-up for pain and satisfaction. The increase in whole-blood cobalt concentration and the reduction in HTD did not affect the HOOS. No correlation was found between either type or size of ALTR and pain or satisfaction scores at 5 years. All patients showed good HOOS score throughout the 5-year follow-up.

## Discussion

We studied the reduction in head–tip distance (HTD) of a modular hip system and compared it with the HTD reduction of a standard hip system. The HTD reduced for the modular group at a rate of 0.15 mm/year. At 8-year follow-up, this HTD reduction was 1.2 mm. We interpret the reduced HTD as a varus deformation in the neck–stem junction. The modular neck–stem deformation correlated with the level of cobalt concentration, and the revised stems showed signs of corrosion. We therefore suspect that the HTD reduction was caused by corrosion at the neck–stem interface, probably in combination with a deformation of the softer stem titanium alloy (TMZF) under the load of the harder neck, made of CoCr alloy (Vitallium).

The increased modification possibilities of modular stems with different neck options have previously been claimed as valuable for ROM, soft tissue balance, and to minimize leg length discrepancies (Barrack 1994, Jones 2004, Archibeck et al. 2011, Srinivasan et al. 2012). The RSA stem migration data showed that the ABG II proximal fit stem, in both the modular and non-modular version, is stable in its resting position after an initial slight subsidence and retroversion within the 3 first postoperative months (Kiernan et al. 2020). There have, however, also been reports on disadvantages related to the additional neck–stem interface when using modular stems. Some have reported fractures of the modular neck (Atwood et al. 2010, Wright et al. 2010) and others have reported ALTR to the metal debris caused by corrosion related to titanium–cobalt–chromium interfaces in modular stem junctions (Duwelius et al. 2010, Gill et al. 2012, Walsh et al. 2015). Pivec et al. (2014) evaluated 202 ABG II modular stems and reported a 3% revision rate up to 2-year follow-up for reasons unrelated to corrosion and 30% revision rate because of corrosion-related symptoms before 2 years. Restrepo et al. (2014) reported in 195 patients 13% revisions at 2-year follow-up for the ABG II modular stem.

8 of our 50 patients with modular stems had been revised 8 years after surgery. Although only 1 of these revisions was directly related to pain in association with corrosion at the neck–stem junction, another patient already had severe ALTR with groin pain before the incidence of PPFF, which led to its revision. The revised necks have all been firmly attached to the stem body, and they did not seem to be loose. A high force was needed to separate the necks from the stem. This high rate of revisions has raised general concerns, and Stryker recalled the ABG II modular stem in June 2012 when concerns arose due to the potential for corrosion at the neck–stem junction.

We are the first to report on the steady rate of HTD reduction in ABG II modular stems, and we are not aware that this phenomenon has been described for any other modular hip stem design before. We found that the modular ABG II stem releases more metal ions into the surrounding tissue, compared with the standard ABG II. This seems to be the case for all modular stems and is confirmed by other studies (Pivec et al. 2014, Kwon et al. 2016, Nawabi et al. 2016). We did not find a correlation between elevated metal ion levels and type of ALTR in MRI as reported in other studies (Cooper et al. 2013, Walsh et al. 2015, Kwon et al. 2018), nor did we find a difference in ALTR between the two study groups or a relation between ALTR and clinical results.

The slow deformation caused by the ongoing corrosion process might not be so harmful in itself, but how long can it continue? We are as yet unable to draw any conclusions regarding the outcome in the long term. It is hoped we shall see a continued leveling out of whole-blood ion concentrations in future follow-ups. This might be an indicator of a steadier state at the neck–stem junction. A far worse scenario is continuing varus deformity of the stem with modular-neck fractures or dislocation of the neck–stem junction. We therefore will continue to follow these patients with RSA and metal ions in blood and, when needed, with MRI.

A limitation of this study is that we lack patient-reported outcome measures for the 8-year follow-up. It was an administrative mistake that these questionnaires were not sent out for the modular stem patients at 8 years, but we will continue to do so in later follow-ups.

Our study is unique in the sense that we measured the HTD with RSA, and we suggest it as a valuable tool for measuring the integrity of a modular implant. If we had been specifically looking for this phenomenon, we would have noticed the difference within the first 2 years after surgery.

In conclusion, in this modular stem design, there is a corrosion-related release of cobalt ions in particular, with a correlated progressive varus deformation of the neck–stem connection. This does not, however, seem to correlate with ALTR, and up to 8 years we have not yet seen a definite clinical problem with neck deformation, but further follow-up is needed.
